# Using Video Processing for the Full-Field Identification of Backbone Curves in Case of Large Vibrations

**DOI:** 10.3390/s19102345

**Published:** 2019-05-21

**Authors:** Marco Civera, Luca Zanotti Fragonara, Cecilia Surace

**Affiliations:** 1 Department of Mechanical and Aerospace Engineering, Politecnico di Torino, 10129 Turin, Italy; marco.civera@polito.it; 2 Centre for Autonomous and Cyber-Physical Systems, Cranfield University, Cranfield, Bedford MK43 0AL, UK; 3 Department of Structural, Building and Geotechnical Engineering, Politecnico di Torino, 10129 Turin, Italy; cecilia.surace@polito.it

**Keywords:** nonlinear dynamics, video processing, backbone curve, noncontact measurements, geometric nonlinearity

## Abstract

Nonlinear modal analysis is a demanding yet imperative task to rigorously address real-life situations where the dynamics involved clearly exceed the limits of linear approximation. The specific case of geometric nonlinearities, where the effects induced by the second and higher-order terms in the strain–displacement relationship cannot be neglected, is of great significance for structural engineering in most of its fields of application—aerospace, civil construction, mechanical systems, and so on. However, this nonlinear behaviour is strongly affected by even small changes in stiffness or mass, e.g., by applying physically-attached sensors to the structure of interest. Indeed, the sensors placement introduces a certain amount of geometric hardening and mass variation, which becomes relevant for very flexible structures. The effects of mass loading, while highly recognised to be much larger in the nonlinear domain than in its linear counterpart, have seldom been explored experimentally. In this context, the aim of this paper is to perform a noncontact, full-field nonlinear investigation of the very light and very flexible XB-1 air wing prototype aluminum spar, applying the well-known resonance decay method. Video processing in general, and a high-speed, optical target tracking technique in particular, are proposed for this purpose; the methodology can be easily extended to any slender beam-like or plate-like element. Obtained results have been used to describe the first nonlinear normal mode of the spar in both unloaded and sensors-loaded conditions by means of their respective backbone curves. Noticeable changes were encountered between the two conditions when the structure undergoes large-amplitude flexural vibrations.

## 1. Introduction

Thin, flexible beam-like structures are ubiquitous in nature as well as in man-made artefacts. Their theory is used to model the behaviour of components such as compliant mechanisms, mechanical flexures, propellers, helicopter rotor blades, spacecraft antennae and wind turbine rotors. Even tall buildings, large span bridges, airplane wings and other large structures can be approximated by these models for some specific tasks, to name a few.

Two major difficulties arise for the experimental investigation of lightweight flexible beams’ dynamics. Firstly, even small amplitudes of the input forces may cause large displacements and eventually induce the related nonlinearities. That makes the basic assumptions of Linear Modal Analysis (LMA), which is arguably the most important tool in the dynamical characterisation of structures, to fail. Secondly, slender elements are, by definition, very sensitive to changes in mass and stiffness, thus hindering the use of physically-attached sensors, such as accelerometers. To adequately address the problem, noncontact nonlinear modal analysis (NLMA) would be required.

One of the main limits to NLMA diffusion is that it is, in the most practical case, not cost-effective: Most standard models can be linearised with good approximation, demanding very limited effort. Whenever this simplification does not hold anymore, the general reception from regulators and practitioners is to define limited areas of application, and simply to not allow the mechanical system to operate in conditions where it can undergo unpredictable behaviours, restraining its use. However, this is not always feasible; and even when it is, it is rarely an optimal solution. The issue is particularly relevant for the aerospace industry. Next-generation airplanes will require, for both military and civil purposes, more and more manoeuvrability to achieve efficiently their aims, such as reduced fuel consumption [1]. State-of-the-art wing designs are increasingly flexible, thanks to light-weight innovative materials and high aspect ratios, and thus also inherently less damped and more efficient. For example, the recent Airbus A350 and Boeing 787 Dreamliner have both much more lightweight structures than their predecessors thanks to the extensive use of composite materials. The industrial trend is to further exploit positively the increased aerodynamic performance of more flexible wings. However, these new prototypes are not free from issues, as predicting and avoiding undesired resonance effects becomes harder. An example [2] is the one of aileron deflection during a standard roll manoeuvre to cause resonance with one or more of the wing’s aeroelastic lower modes, thus altering the desired motion.

Major strides were made in recent years for the nonlinear characterisation of aeronautical structural components (e.g., [3,4,5,6]) to better understand the inherent dynamics involved and to predict them with good precision; a complete overview can be found in the classic review [7] and in its recent update [8]. However, to the best of the authors’ knowledge, most of the well-recognised experimental works in this sector resorted to physically-attached accelerometers or laser Doppler vibrometers (LDV). For instance, [9] stated that the weight of the sensor, located at the beam tip, was uninfluential; the same cannot be said in the experimental case here presented. Indeed, MEMS sensors are generally the default choice for vibrational analysis, and new prototypes are continuously developed [10]. Recently, the use of piezoelectric patches glued to the structure, widely used for vibrational analysis and control [11,12], was also proposed for experimental nonlinear modal analysis [13]; however, patches’ mass, while distributed over a larger surface than in the case of accelerometers, is still not negligible for thin structures. No attempt to use contactless measures of the large deflections’ displacements for NLMA seems to have ever been performed via video recording, while optical methods are starting to be widely used for modal analysis at the current state (e.g., [14,15]).

In the case here presented, the aluminum spar of a prototype high aspect ratio (HAR) aircraft wing has been investigated. The prototype was designed and manufactured in the framework of the BEARD project [16] at Cranfield University. The experimental campaign here reported aims at extracting a specific nonlinear modal feature, the so-called backbone curve, for the first nonlinear normal mode (NNM), at the highest amplitude level possible, using a noncontact optical measurement system. Thus, high-speed, high-quality video recordings and video post-processing techniques were applied to the free decay response from a nonlinear resonance. Promising results were achieved.

The rest of the paper is organised as follow. In Section 1, the theoretical context of this research is described. In Section 2, a brief recall about the theoretical background of NLMA in general and of backbone curves in particular is introduced for the specific aim of this research. The description of the structure investigated, the experimental setup and the research context are reported in Section 3. The video processing approach for the characterisation of the nonlinear dynamics is presented in Section 4. Results are presented in Section 5, and Conclusions follow.

## 2. Large-Amplitude Flap-Wise Bending Oscillations

In this Section, some hints about the nonlinear theory of deformation of wings are reported to provide a minimum context. A much more exhaustive discussion can be found, for instance, in the Section 6.6 of [17].

**Figure 1:**
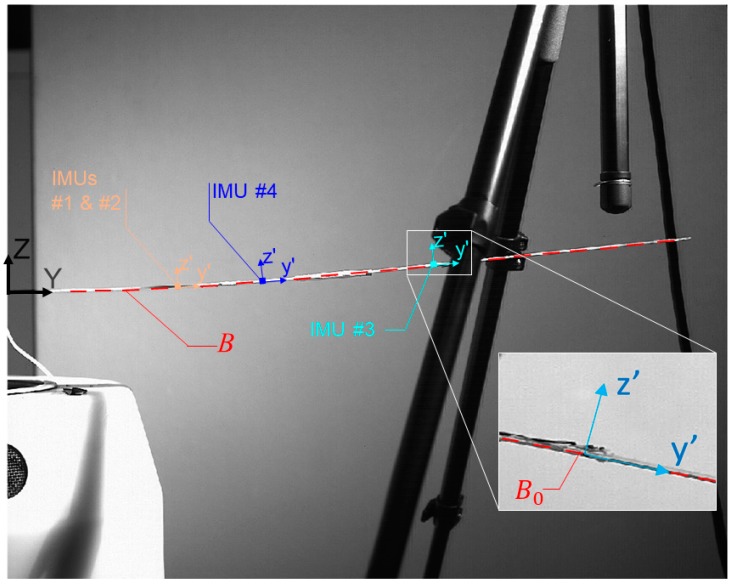
Global and local reference systems (RS). Note that the accelerometers’ own {*O′,x′,y′,z′*} RSs (zoom box, bottom right corner) are not always corresponding to the global {*O,X,Y,Z*} RS but always consistent with the direction of the restoring force. Deformed (B(t)) and reference ($B_{0}$) configurations, the latter one deflected under its own and sensors’ weights, are highlighted.

Let us consider the beam-like wing spar of interest, reported in side view in Figure 1. For clarity’s sake, let us also define a fixed, global reference system (RS) {*O,X,Y,Z*} located in the elastic centre of the root wing section, distinct from the locally-defined, non-inertial RSs {*O′,x′,y′,z′*}, which translate consistently with the cantilevered spar. Being the spar cross-section rectangular everywhere, the elastic centre is always coincident with the centroid due to its double symmetry. The structure is monolithic, nonprismatic (the beam has constant thickness but is tapered horizontally, i.e., the cross-section width changes along beam main axis with a pointwise continuous rate), with fixed-free boundary conditions and base-loaded for the mean of this study; it can also be approximated as unshearable. Its material (Aluminum 7068 alloy) can be assumed as linear isotropic, and its properties are constant in time and uniform everywhere.

The wing stress-free, unloaded reference configuration $B_{0}$ is defined at equilibrium under its own weight and with no external static or aerodynamic loadings of any sort (no base acceleration, airstream or added masses) applied. It is crucial to remind that $B_{0}$ has a non-negligible curvature due to its own weight under gravity. During large oscillations, the deformed configuration B(t) cannot be confused with it, differently from what is commonly done for small vibrations.

Apart from small and very small axial elongations, the only large motions that the structure can undergo are flexure about the two principal axes (i.e., flap-wise and chord-wise) and torsion around the elastic axis [18,19]. For the intent of this study, torsion was not considered. Moreover, for wings with high aspect ratios, chord-wise (that is to say, side-to-side) bending is very small in comparison to the deflections along the direction with a smaller moment of inertia; therefore, it was also neglected here. In doing so, it is possible to reduce the problem to a two-dimensional form, fully defined in {*O,Y,Z*}. This makes single camera recordings sufficient to capture the dynamics of interest, if all of its points of interest fall into a single focus plane. Even more than the bending along the *X*-axis, axial stiffness is orders of magnitude larger than the flexural one; thus, as mentioned above, elongations are generally overlooked, with the Euler–Bernoulli (EB) theory assuming the beam as inextensible. Nevertheless, these minimal changes in beam axial strain are exactly the foremost cause of the nonlinearities which are of interest here; a deeper discussion can be found, for instance, in [20].

Let us consider a harmonic external driving force, with a (relatively) very high energy content, transmitted by the moving clamp. The nonplanar dynamics which arise from this make the continuous system weakly nonlinear. For this kind of very slender, highly-flexible beam-like structures, three main sources of nonlinearities arise during large oscillations; obviously, the higher the aspect ratio of a lifting surface, the larger the transverse flap-wise displacements it will be subjected to. 

The primary source is due to the so-called *geometric nonlinearities*, which are described later in detail. The secondary source is due to inertia nonlinearities [21]. Finally, also air drag, which can be modelled as a quadratic damping component, may be considered for completeness [22,23], even if its estimation is arguably more subject to uncertainties. The first two factors are concurrent and balance each other, as they produce, respectively, hardening and softening nonlinear behaviours for an increasing amplitude of the input, with comparable orders of magnitude for large deflection dynamics [24]; nevertheless, geometrical nonlinearities are known to be predominant on the first nonlinear mode of a structure similar to the one inspected here [25] and are therefore of major interest in this ambit.

When the linear threshold is exceeded, two related and intertwined practical problematics arise: (1) Estimating the frequency of the (nonlinear) resonance and (2) estimating the amplitude and direction of the correlated motions. Quite interestingly, large deflections have been most extensively studied for static load cases rather than from a dynamical standpoint, while geometric nonlinearities are investigated predominantly from a frequency content or a phase space point of view. The literature regarding the large oscillation dynamics is somehow limited, heavily relying on the classic works of Hermann [26], Yamaki [27], Mei [28] and Wah [29], which was also one of the firsts to point out the correlation between the unforced large vibrations and the nonlinear continuation of normal modes [30], few years after their very first definition by Rosenberg [31] (the topic is better covered in the next Section).

For this kind of structures where mobility is given by structural members’ flexibilities rather than rigid-body joints, the term *compliant mechanism* [32] is used. If excessive deflections cause the strains to invalidate the commonly accepted infinitesimal strain assumption, the transverse displacements of an elastic prismatic beam cannot be computed according to the classic Euler–Bernoulli beam theory, due to its own basic assumptions. This derives from the EB theory neglecting the square of the first derivative in the curvature and assuming—as previously mentioned—the beam’s inextensibility [33]. Thus, the estimated transverse displacements are highly overestimated and unrealistic. A more exact solution is not a trivial task, often subject of research (see, e.g., [34]). Considering the second-order elements in displacement neglected by the EB beam theory leads to the full Green–Lagrange strain tensor, the terms of which are the classic linear ones plus their mixed quadratic components, described by the finite strain theory [35]. These nonlinear components of strain, in turn, cause a subsequent increase of tensile stresses known as *stress stiffening*. It is possible to reduce the complexity of the task by making proper assumptions; this produces still nonlinear, yet simplified kinematical models, such as the Föppl–Von Kármán plate [36,37,38], much easier to define and widely used in practice (the previously-cited works of Hermann and Yamaki both resorted to Von Kármán equations, while Mei and Wah applied the similar Berger’s hypothesis [39]). However, these nonlinear effects, which can be modelled as higher-order terms of additional stiffness, cannot be reduced to an explicit form; therefore, their dynamic response can be only numerically simulated with incremental and iterative approaches. 

Since the total stiffness increases with the deflection, this behaviour can be represented in function of the amplitude of the external driving force applied. This amplitude–frequency graph is better known as the *backbone curve* [40,41] and is addressed at the end of the next Section.

## 3. Nonlinear Modal Analysis in Case of Geometrical Nonlinearities

LMA is extensively used for the dynamic characterisation of experimental structures, both using input–output (experimental MA) or output-only (operational MA) methods [42]. Since the widespread commercialisation of digital Fast Fourier Transform (FFT) analysers, the discipline has become a common tool to validate, understand and monitor models for both experimental research and common industrial practice; the theoretical background has for decades been mature and well-defined [40,43].

However, almost all real-life systems—both natural and man-made ones—are somehow nonlinear, and their dynamics are dominated by additional terms (always present but negligible in the linear approximation), which make their behaviour more difficult to model than for their linear equivalents [44]. This affects drastically the system response, as it is immediately appreciable in the frequency domain [45,46], where steady-state and transient frequency response functions (FRFs) are no more coincident [47]; the typical behaviour of the response in frequency domain is a right- or left-leaning steady state nonlinear FRF, depending on if hardening or softening is prevailing when the energy content of the forced system increases. In these cases, white Gaussian noise or continuously-swept sine inputs cannot be used [48], due to modal coupling; they do not provide useful insights in the system dynamics, even if some attempts have been performed in the last years [49]. A stepped sine sweep procedure is therefore necessary to focus on the steady-state response of the system and to follow properly its energy-dependent resonant frequencies [50]. 

The whole description of nonlinear modal analysis would be too long to be condensed here; hence, only the main concepts and definitions used will be briefly summarised; interested readers can find an exhaustive discussion in the book of [41]. The main issues of NLMA are that:

1. No definitive, comprehensive theory has been yet defined and commonly accepted;
2. The response of a nonlinear system is almost always much more complex than its linear counterpart (in particular, it is no more input-independent);
3. ‘Nonlinear’ is a vast and often ambiguous definition, as it only characterises what a structure is not, and not what it actually is.

It is thus better to spend a moment to properly define the particular case of interest here.Two main competing definitions currently exist in literature: The already-mentioned Rosenberg NNMs [31] and the Shaw–Pierre NNMs [51]. The first definition is the most straightforward extension of the classical definition of a linear normal mode, as synchronous and periodic *oscillations* reach their respective maxima in all modal coordinates at the same time. The second definition describes a nonlinear mode as an invariant *manifold*, which is locally a graph over the two-dimensional modal subspace of the linearised equivalent system. Interestingly, if one drops Rosenberg’s requirement of synchronism (as often done), conservative Shaw–Pierre NNMs can be considered as the surfaces where Rosenberg NNMs start and remain constrained in the phase space. Throughout the years, these two original definitions have been enriched, reformulated, generalised and/or relaxed by several authors; for a wider discussion, one can refer to some classic lectures such as [52,53,54,55,56]; some extensive overviews can be found in [57,58]. Other similar, related definitions have been also proposed (e.g., [57]). In particular, [58] recently put forward a variant of the Shaw–Pierre definition, the spectral submanifold, used to extend the useful Shaw–Pierre–Rosenberg NNMs graph relationship in the case of nonconservative and non-autonomous systems and to assure the uniqueness of the so-obtained invariant manifolds (which is not automatically achieved within the classic Shaw–Pierre definition [59,60]). 

Here in this work, the free decay response from phase resonance was experimentally investigated to extract the backbone curve of the first NNM. Since the captured motion started on an invariant manifold and then remained unmolested by external forces (with the exception of gravity), it is constrained to remain on that specific manifold from which it generated. Thus, it is possible to extract from it the modal information which defines the motion itself (i.e., the Rosenberg NNM) and the phase–space surface it belongs to (i.e., an invariant manifold, the Shaw–Pierre NNM). To this aim, here an NNM is defined (by relaxing the Rosenberg’s definition) as a near-equilibrium, periodic or quasiperiodic motion, analytically describable by the closure of a multifrequency solution of the nonlinear damped system of interest. 

The backbone (BB) curve represents the skeleton of the nonlinear response in frequency domain for the nonlinear extension of a given *j*-th mode of the investigated structure. Differently from the linear conditions, this one-dimensional geometrical locus of damped natural frequencies is not a straight line but presents a curvature, as the frequency increases (hardening) or decreases (softening) with the increasing input amplitude. The BB curve allows quantifying the occurring nonlinearities in the condition of interest; also, it permits to investigate the modal energy exchange due to superposing and/or competing nonlinearities. Importantly, it can be fully extrapolated from a free decay from resonance [61], as the transient response is known to contain all information about the underlying fundamental features of the investigated dynamical system, it being linear or nonlinear. This can be simply achieved by plotting the instantaneous amplitude of the recorded signal versus its instantaneous frequency; this method is known as the resonance decay method (RDM) [62,63,64] and has already been successfully applied to an experimental case study of large displacements inspired by the aerospace industry [65], even if, in that case, the output acquisition was performed by means of physically-attached sensors. The theoretical foundations of the method are based on the nonlinear extension of phase resonance testing for the force appropriation of a single NNM, which in turn remains confined to its spectral submanifold of origin during the following free decay, thanks to the invariance principle. This discloses the energy dependence of the NNM with the need only of simple time–frequency analysis techniques. These concepts are all well explained and described in detail in [66]. The main novelty proposed in this work is the execution of this procedure contactless, by coupling it with an optical acquisition technique, to obtain spatially dense information from the unmolested mechanical system investigated.

## 4. Experimental Setup

The specifics of the HAR wing spar studied are reported in Table 1. This beam-like structural component is supposed to completely absorb the aerodynamic loads applied to the air wing, as its skin was realised to minimally affect its dynamical response [1]. Differently from what is expected from a standard longeron, it can undergo also torsional rotations around its main axis.

The spar dynamic response at steady state was found to be always dominated by the undamped linear mode whose resonance frequency is closer to the driving force only frequency component. None of the structure’s modes were found to be involved in an internal resonance with the other ones, and they are all well-distanced. Thus, all the other modes, being not directly nor indirectly involved, decayed much faster due to damping [67,68].

Being that the tests were carried out over several days, a daily check on linear natural frequencies of the spar was performed, by applying a random noise input at low amplitude. This was intended to ensure that no fatigue damage could alter the structural dynamics investigated. The values showed no substantial frequency shift for any of the first three modes. Minor fluctuations (up to three decimal places) were discovered, most probably due to the measurements performed not in a temperature and humidity-controlled room; however, no clear trends or permanent changes were detected.

**Figure 2:**
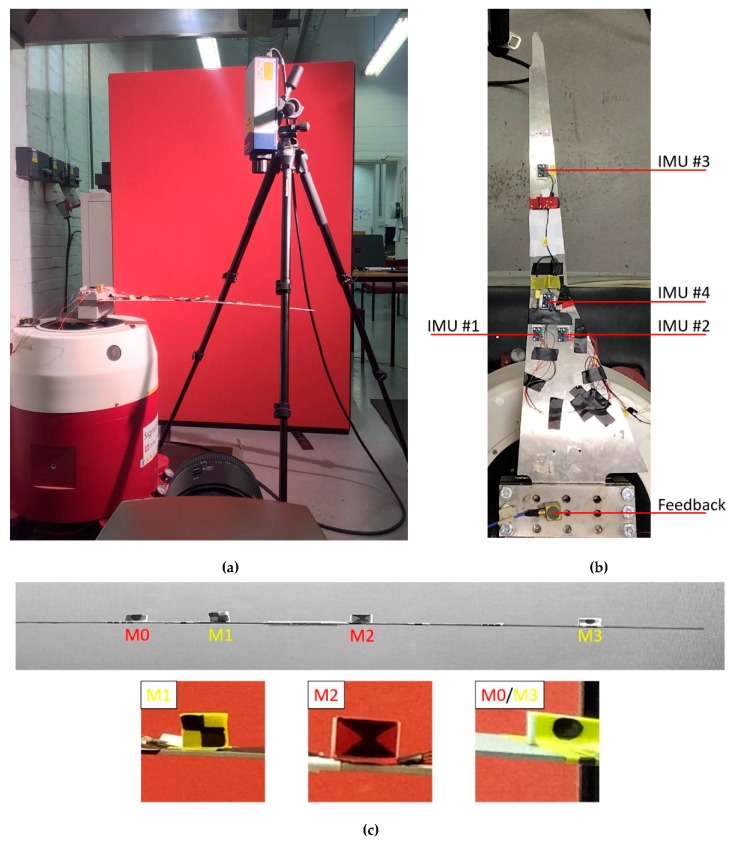
(**a**) Experimental setup: Modal shaker, high-speed video camera and laser velocimeter. Laser velocimeter was used to check the frequency at a given point relatively close to the spar tip. A solid red curtain was used to provide a uniform background. An open-faced lighting fixture with tungsten light source was used to provide strong illumination, near perfect colour rendition and to avoid nefarious effects related to AC illumination. (**b**) Top view of the four inertial measurement unit (IMU) sensors for the loaded condition, of the feedback accelerometer and of the several markers attached on the top surface of the spar. Markers M0 and M3 were removed during tests with IMUs attached. A red laser dot is also clearly visible. (**c**) Close-up of the air wing spar trailing edge, with no sensors but all four markers attached. M0, not shown, has the same pattern as M3 but on a red background. The trailing edge of the spar was completely included in the plane of focus.

The experimental setup is pictured in Figure 2. Recordings were performed with an Olympus ^®^ I-speed 3 ™ high-speed camera, pointed to the spar trailing edge. Polytec ^®^ OFV-505 ™ single point LDV was used for calling the phase quadrature of the output signal (defining so the phase resonance of the system). The input was applied as an external acceleration at the clamped end ($a_{b}$) by means of a Data Physics^®^ Signal Force™ modal shaker and its DP760™ close-loop control software. The software was set to increase the input frequency of 1 mHz at any step and to dwell until steady state was reached before sweeping to the next one. The duration of the frequency steps was not constant; an automatic settling time option was used, considering 1% of tolerance between consecutive periods of the output.

Unloaded and sensor-loaded configurations were studied, to address specifically the effects of sensors’ added masses on the nonlinear response of the system. In the latter case, four Raspberry PI^®^ inertial measurement units (IMUs) were used. The sensors were located at Y=200, 250 and 450 mm from the clamped end and numbered according to their output channel (Figure 2b). The whole acquisition apparatus consisted of the 4 IMUs, each one weighing 2 g, cables, tape to attach the cables to the structure (to avoid detachment and confusing, unrelated motions in video recordings due to their cluttering) and double-sided adhesive foils to attach sensors to the spar extrados, for a total weight of 11 grams. This has been proven here and in previous studies to not affect the quality of measured accelerations, while being minimally invasive. Other specifications about the Raspberry PI ^®^ IMUs utilised for the air wing spar here investigated are reported in [69]; for completeness’ sake, the most relevant technical details about them and the rest of the instrumentation are reported in Table 2. 

Two markers were attached on the spar top surface, at Y=260 and 390 mm. They were labelled (in the same order) as marker #1 and #2. For the following tests without sensors, markers #1 and #2 were left, and two more were added, for a total of four, respectively, at Y=180 mm (marker #0) and at Y=600 mm (marker #3). That makes the ‘unloaded’ condition actually loaded with four larger markers, attached on the spar extrados, plus other pieces of paper and tape. However, the overall effect on the overall mass is basically null, as reported in Table 3, each marker’s mass being less than 0.07 grams.

The high-speed camera was calibrated before starting the video acquisitions. Graph paper was attached on the beam profile to have a constant double check. Due to the internal memory limitation of the camera to 4897 frames (with 1280 × 1024 pixels per frame), any increase in frame rate (i.e., the number of frames per second) is inversely proportional to the maximum duration of the video. To capture with high fidelity the decay response, several values of sampling frequency were tested; wherever not specified differently, $f_{s-VIDEO}=500$ Hz was set. Mainly because of motion blunder, the selected value was much larger than the minimum required by the Nyquist criterion. This limited the effectiveness of the study, as it was not possible to fully include the whole decay. Thus, the focus was given to the initial part of it, at larger amplitudes. 

The study here reported was restricted to moderately large deflections, as very large transverse displacements would have caused the spar to snap under bending. To be non-unrealistically large, the angular deflection can never exceed the beam breaking point; this limit is generally assumed to be a vertical component of motion at the cantilever beam most restricting point (the tip) equal to 30% of the beam effective length. This is consistent with what is usually seen in ultimate-load wing-up bending tests, where the wing is forcedly bent upward until it reaches collapse; for instance, the Boeing 787 Dreamliner airframe ZY997 reportedly supported a vertical tip displacement of approximately 25 ft (circa 7.62 m) before rupture, which is roughly more than 25% of its 98.5 ft (circa 30 m)-long semispan. This limitation was also intended to avoid any fatigue effect and permanent damage on the specimen, hampering its use for following tests. Thus, the base acceleration $a_{b}$ was set to 55 milli-g, equal to 0.5394 $m/s^{2}$.

## 5. Video Processing Application of the Resonance Decay Method

Let the deflections, velocities and accelerations transverse (flap-wise) to the beam main axis be $w,\dot{w},\ddot{w}$. Most commonly, accelerations are recorded through physically-attached accelerometers, then numerical integration techniques are applied to achieve an estimation of velocities and displacements, i.e., $\hat{\dot{w}}$ and $\hat{w}$. If the actual motion happens to be lying on a straight line, laser vibrometry is a common tool to measure vibrations, recording directly either velocities or displacements. Both accelerometers and LDV are standard practices, and their advantages and limits are well known [70].

**Figure 3:**
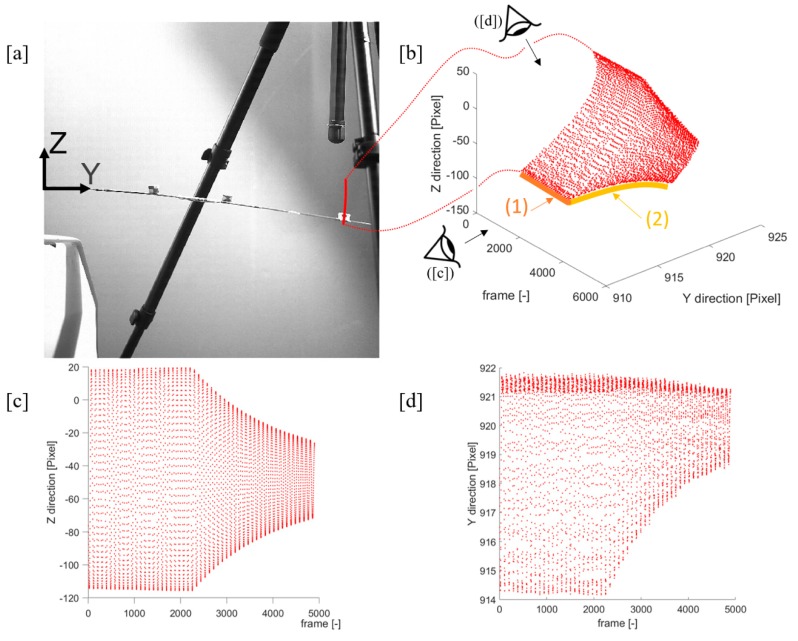
From top left: (**a**) Example of results superimposed to the target frame; (**b**) time history of displacement (in pixels): A free decay (2, yellow arrow) after dwelling (1, dark orange arrow) is reported; (**c**) horizontal component of the 2-D motion in focal plane; (**d**) vertical component of the same. Acquisition rate: 1000 fps.

In the case of large deflections, the motion cannot be anymore assumed as a straight vertical line, and its curvature must be accounted for (Figure 3); thus, nontracking LDV measurements are not correct. This is naturally adjusted by physically-attached sensors, as they record according to their own RS, which is coincident to the locally-defined (cross-section-wise) roto-translated wing RS {*O′,x′,y′,z′*}. However, it is not possible to easily extrapolate from their data the actual motion with respect to an external, fixed, time-invariant reference system. Moreover, both accelerometers and single-point LDV only produce point-wise measurements, whilst a single video camera allows producing full-field, span-wise information among the frame two-dimensional space and is therefore well suited for the characterisation of complex phenomena. Nevertheless, video acquisitions produce very large tensors of data, equal to $\left(l\times h\times N_{f}\right)$, where *l* is the frame width, *h* its height and $N_{f}$ represents the number of frames captured. Moreover, most of the standard coloured formats are made up of three elements per pixels (such as hue, saturation, lightness, or alternative triplets), tripling the amount of data. Generally, most of the information contained is of little or no use, making optical techniques as comprehensive as they are redundant. Thus, the extraction and synthesis of useful and reliable data is a major aim in measurement technological advance.

The main assumption is that the transverse motion is all contained in (or can be approximatively projected on) a properly selected focal plane, perpendicular to the camera line of sight and with a given, fixed distance from its lens. A preliminary study about the comparison of LDV, accelerometers and the video point tracking technique utilised here can be found in [69]. The optical measurement approach, based on the speed-up robust features (SURFs; [71]) extraction frame-by-frame, is also described more in depth there, with major technical details on the implementation of this technique and a discussion on its feasibility for the acquisition of large deflections.

The procedure here applied requires the force appropriation of a single NNM per time, using a harmonic force with constant amplitude and stepped increased frequency. The desired resonance condition was checked by means of the phase quadrature between the input and the output signals, recorded by the feedback accelerometer and the LDV, respectively. As mentioned above, due to the large deflections, nontracking laser suffers from a sliding effect of its point of application; this makes its amplitude measurements incorrect. Nevertheless, the physical zero crossings happen at the same point at any period, if the laser were originally focused on the beam in the resting position; and an area around of the zero (quiet position) can always be linearised. Hence, it is still possible to use the laser to correctly measure the frequency of the vibration at its targeted point. Thus, it can be used for calling the phase resonance, when input and output signals have a phase-lag of 90°. This was indeed the strategy used to decide when to switch the modal shaker off and the camera on and to start recording to perform the resonance decay method via video recording.

**Figure 4:**
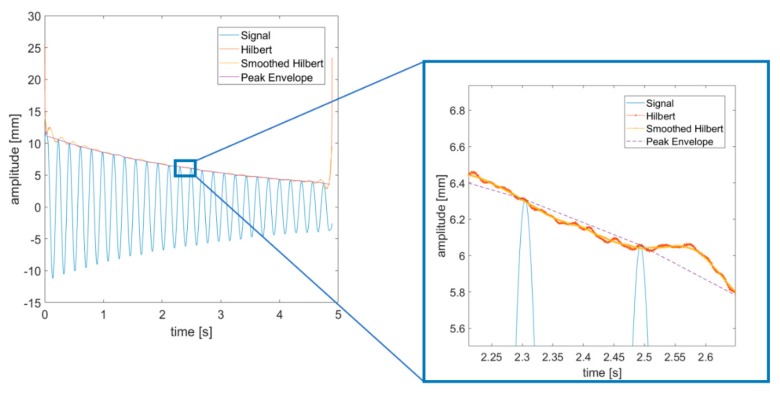
Estimation of instantaneous amplitude (to the left) with a zoom (to the right). Video-acquired data, free decay (Marker #0). Acquisition rate: 1000 fps.

**Figure 5:**
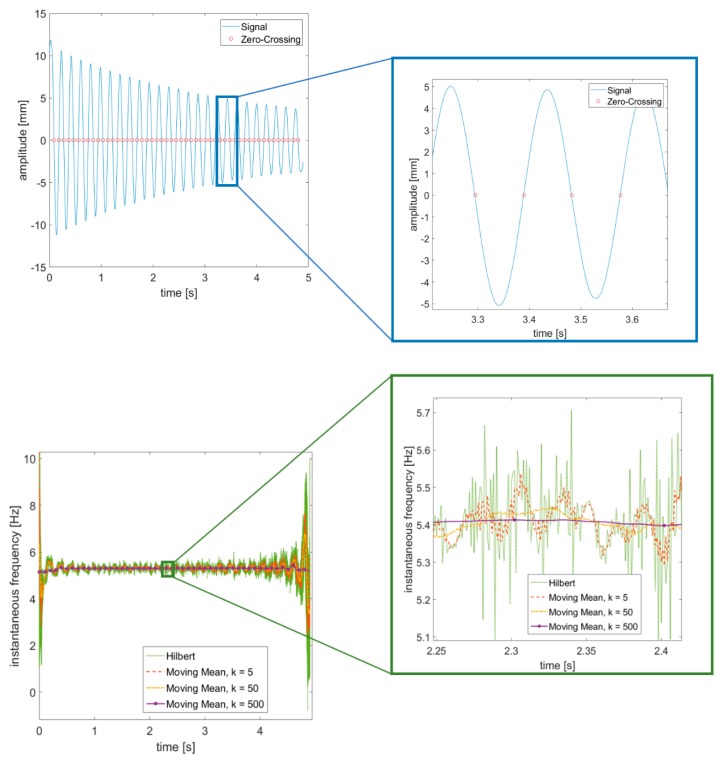
Estimation of instantaneous frequency (top) through zero crossing. Hilbert Transform is also shown for comparison, with a zoom (bottom left and bottom right, respectively). Same example as in Figure 4.

Once the video-acquired time histories (THs) are collected, their instantaneous frequency and amplitude can be assessed by means of zero crossing and peak enveloping, as suggested by [62]. Indeed, both zero crossing (ZC) and peak envelope (PE) are known to outperform the Hilbert transform (HT) in terms of accuracy and robustness to edge effects (some remarkable steps to overcome the implicit issues of HT have been proposed [72], but their application to extract the backbone curves of a mechanical system is more elaborate in terms of signal processing); a comparison is reported in Figure 4 and Figure 5. The ZC method can be easily applied in case of symmetric restoring force (as here), even if some extensions to asymmetric response signals have been proposed very recently [73]. A moving average was applied to smooth out the noisy signals recorded, as again also suggested in [62].

## 6. Results

**Figure 6:**
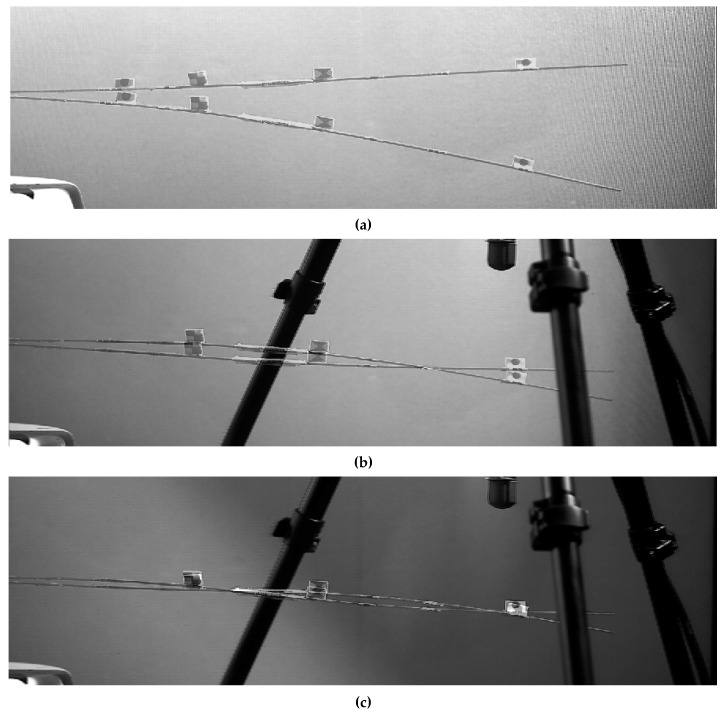
Operational Deflection Shapes corresponding to the first (**a**), second (**b**) and third (**c**) nonlinear normal modes.

All the acquisitions reported in this Section were performed with a frame rate of 500 frames per second (fps). As a preliminary study, the first three operational deflection shapes (ODSs) of the wing were investigated as extracted from the video recordings (Figure 6). They correspond, respectively, to the first, second and third flap-wise flexural modes. However, for the given direction and point of application of the input force, only the first mode experienced transverse displacements large enough to cause relevant geometric nonlinearities for the level of amplitude applied. This can be easily explained as the first bending mode as the larger effective length, equal to twice the free length of the spar ($l_{0}=2l_{tip}$). Thus, the nonlinear component of the modal displacements of the second and higher modes is negligible.

It was confirmed experimentally that the first NNM is much slower to decay than the next ones. For the maximum amplitude input at the clamped base, $a_{b}=0.055\;g$ (0.5394 $m/s^{2}$ in SI measure), the settling time of the free decay (considered as a reduction to the 10% of the amplitude at steady state, T_s10%_; reported in Table 4) is upper bounded to circa 15 seconds for the first nonlinear normal mode; to circa 5 seconds for the second (basically linear) mode; and to no more than a couple of seconds for the third. 

### 6.1. Time Histories Extracted from Video

**Figure 7:**
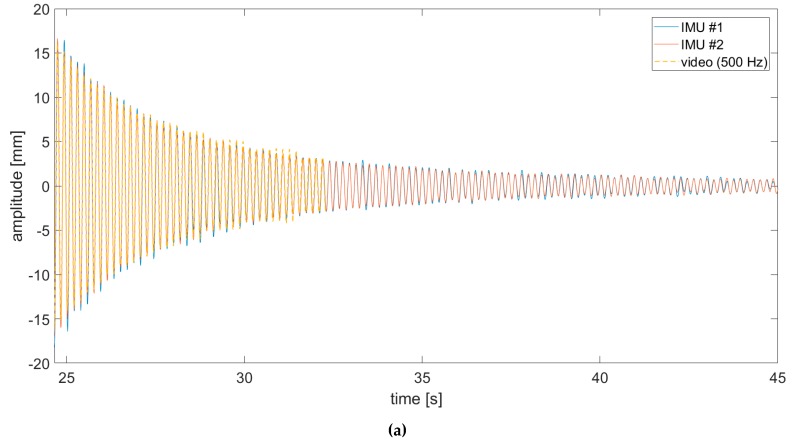

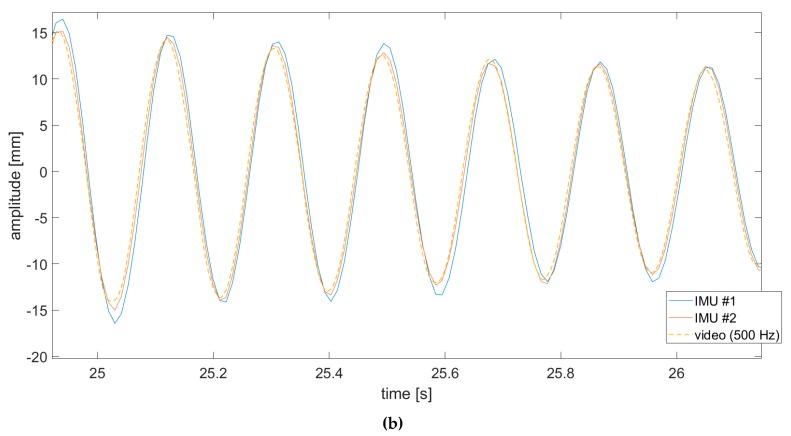
(**a**) Comparison of displacement time histories (video vs. IMUs #1 and #2 acquisitions). (**b**) Zoom on a one-second-long tract.

**Figure 8:**
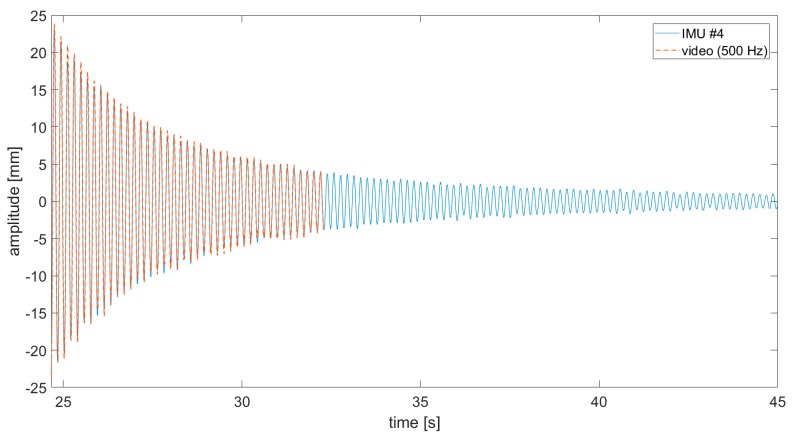
Comparison of displacement time histories (video vs. IMU #4 acquisition).

**Figure 9:**
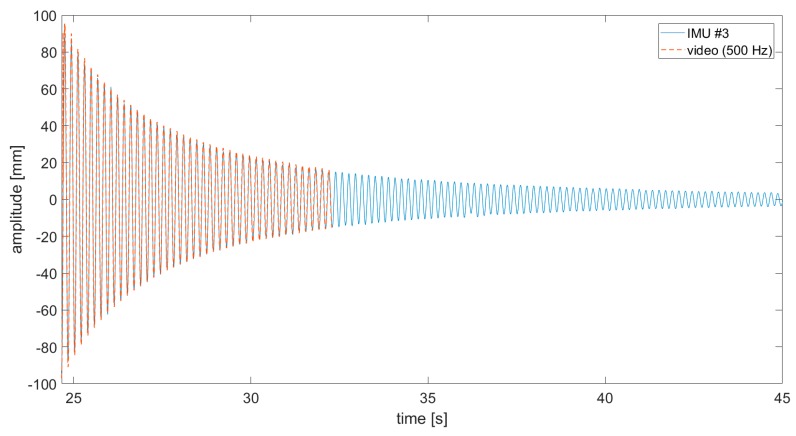
Comparison of displacement time histories (video vs. IMU #3 acquisition).

A comparison between video-acquired and accelerometers’ THs was firstly run on the sensor-loaded configuration. The SURF targets where extracted from subframe pixel regions cropped around the sensors and used for frame-by-frame target detection. To benchmark video-acquired data on IMU recordings, conventional data fusion approaches were used. Data from the physically-attached sensors were sent and collected in real-time to a nearby laptop via a local wi-fi connection. Due to technical limitations on the bandwidth, a maximum sampling frequency of $f_{s-IMU}=100$ Hz was available; that was five times lower than the video frame rate. The sensors were switched on only during the decay. All the recordings were then converted to displacements in millimetres by post-processing the results. Results show a good similarity (Figure 7, Figure 8 and Figure 9; data are superposed to accelerometers’ THs for the duration recorded by the camera).

It was found that the video acquisition, performed on the trailing edge, better approximated the IMU sensor closer to the edge (Figure 7b), suggesting some transient torsional effects at the beginning of the free decay. Note that in all pictures, the zero of the deflection amplitudes corresponds to the position of the wing in resting conditions for that *Y* coordinate along $B_{0}$.

Being the optical method successfully benchmarked against the IMUs, the markers were also used as targets for the feature detection and tracking algorithm, for both the unloaded and loaded conditions. It was empirically observed that, of the several marker designs tested, the solid circle pattern (markers #0–#4) performed better than the checked (marker #1) and the x-shaped (marker #2) ones. This can be specific for the case study here analysed; nevertheless, this is most probably due to the marker detection algorithm being based on the brightness gradient, which is equally sharper in all directions for a circular, solid pattern. Resulting time histories were howsoever appreciable for all markers. The next step presented here involves a useful approximation for the curvilinear motion deduced by the two-dimensional trajectories traced.

### 6.2. Parabolic Local Approximation of Video-Extracted Transverse Large Motions

This subsection deals with the definition of the motion corresponding with the NNM investigated. As mentioned in Section 2, a motion started on a submanifold is constrained within it during a free decay, by the definition itself of a (nonlinear) mode. This is the basis for the RDM method and allows defining the mode associated with the BB curve.

It is known that the exact expression for the curvature of the elastic line under a static, transverse point load applied at the free end can be conveniently rewritten in terms of arc length and slope angle [33]. Thus, the deflection formula assumes the form of complete and incomplete elliptic integrals of the first and second kinds [74]. It is therefore possible to extend the discussion to the beam dynamic response, for large oscillations under a harmonic excitation. These are here assumed to be, at any time, inscribed within an ellipse oblate along the axis that passes through the clamped cross-section and is parallel to the beam axis at that point. Thus, the curvilinear displacement at any cross-section can be computed as the arclength between the beginning and the end points of interest at a given time. The HAR wing here studied is nonprismatic, and the load is applied by means of forced displacement at the clamped end; the first assumption made, therefore, was that the trajectories which better describe the perpendicular motion of the beam-like air wing spar at any cross-section (i.e., its *deflection paths* at any point *Y*) remain elliptical. This assumption was found experimentally to be acceptable for the specific case study of interest.

**Figure 10:**
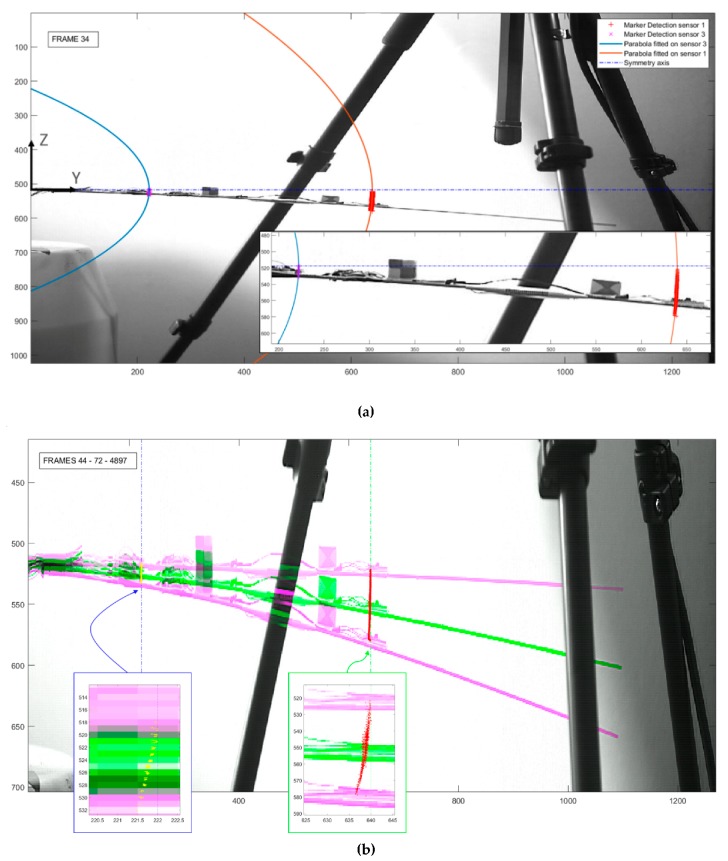
(**a**) Detected targets and the parabolas fitted on them (solid blue and red lines) superimposed to a general frame; the shared symmetry axis is shown as a dashed–dotted blue line. (**b**) Detected targets superimposed to the spar minimum, maximum of the deflection (pink) and to the same spar in rest condition (green). Tangent lines to the two parabolas are shown as dashed–dotted blue and green lines.

For every point along $B_{0}$, the second hypothesis made (confirmed by experimental observations) was that the elliptical transverse motion can be locally approximated around the region of interest with a tangent parabolic trajectory. This is more appropriate than considering a local approximation to a circle with a fixed radius, as commonly done in practice with the so-called *pseudo-rigid-body model* (see, e.g., [75]). The divergence between the ellipse and its local parabolic approximation becomes relevant only at very large deflections, closer to the beam final flexural resistance and not investigated here. Thus, for any point on which the marker detection was performed, a unique parabola, opening to the negative direction of the *Y*-axis, was fitted according to the first NNM (Figure 10). It has been noticed that all the so-defined parabolas are not confocal, but nevertheless, they share the same axis of symmetry; this axis was experimentally seen to overlap with the horizontal line tangent to the spar at the clamped cross-section.

**Figure 11:**
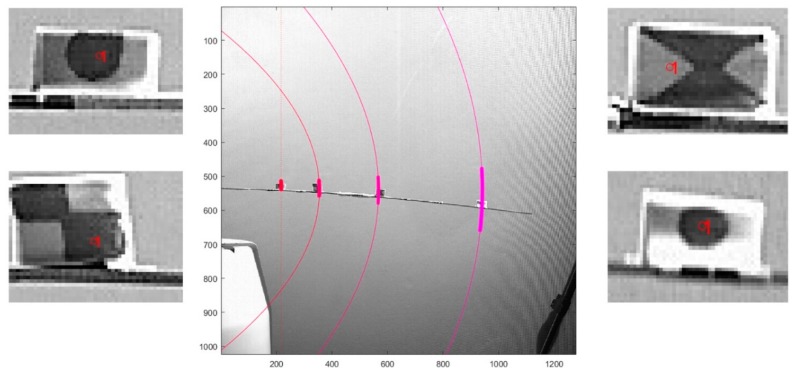
The four markers used in the unloaded configuration; specific features followed frame-by-frame indicated by a red circle. Parabolas fitted over markers #1, #2 and #3 are shown as solid curves in shades of purple. Transverse displacements of marker #0 were too small to successfully fit a parabola on them, but the small motion might be assumed as a straight vertical vibration (dashed red line) with negligible error.

Let us consider the general equation of the parabola $Y=p_{1}Z^{2}+p_{2}Z+p_{3}$ (with $p_{1}\leq 0$ opening to the left, i.e., for negative *Y* values). *Y* being the axis of symmetry, the general form can be rewritten as $Y=p_{1}Z^{2}+p_{3}$. By translating the global RS so that the vertex of the parabola falls on (0,0), it can be further simplified to $Y^{\prime }=Y-p_{3}=p_{1}Z^{2}$. Obviously, for any cross-section close to the fixed end, where perpendicular displacements are small enough to be linear or almost linear, the tract of the parabolic curve becomes so small that it can be approximated to a straight vertical line (see marker #0 in Figure 11, for instance). In this case, the curvilinear motion basically overlaps with its vertical component.

Values of $p_{1}$ for the example reported in Figure 11 are enlisted in Table 5. The goodness of fit is generally very good; the sum of squares due to error (*SSE*), the R-squared coefficient of determination ($R^{2}$), the degree-of-freedom adjusted R-squared coefficient of determination (${\bar{R}}^{2}$) and the root-mean-square error (*RMSE*) are all reported, as well as the degrees of freedom in the error (*DFE*). For Marker #0, the displacements were small enough to linearise the motion; hence, no parabola was fitted over it.

### 6.3. Video-Extracted Backbone Curves

**Figure 12:**
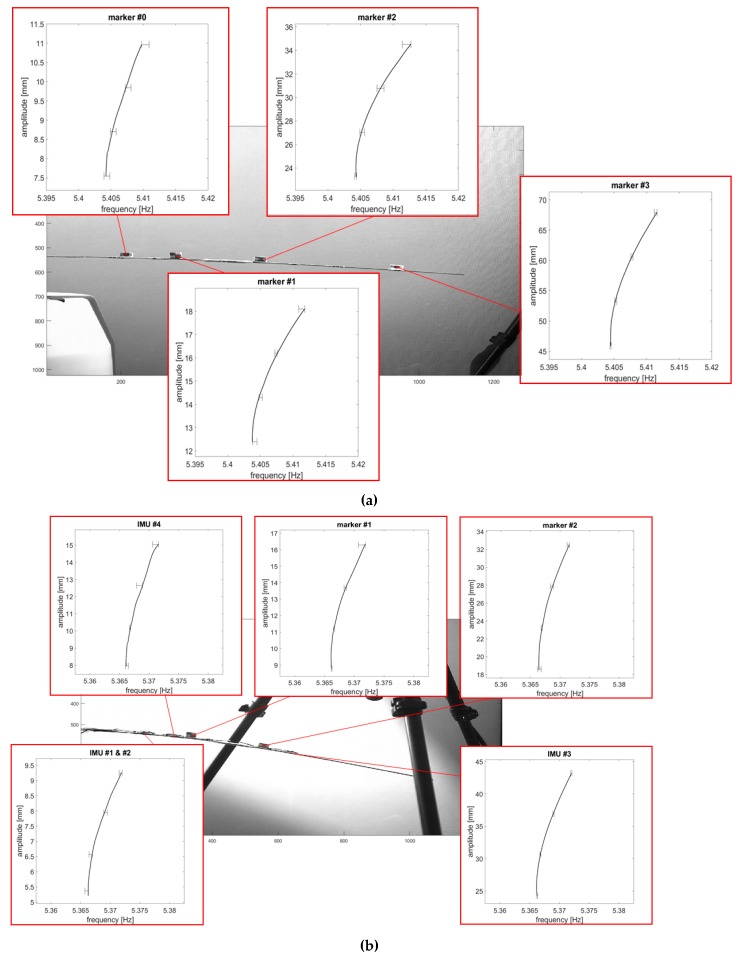
Video-extracted backbone curves: (**a**) Unloaded condition and (**b**) sensor-loaded condition. The portrayed curves come from the first round of measurements; error bars show the range of frequencies for the three rounds at four fixed instants.

The backbone curves were extracted from the free decay of the first NNM for both the unloaded and the loaded structure (Figure 12), excited for the aforementioned value of $a_{b}$ (0.5394 $m/s^{2}$). In the first case, the results for the four markers #0–#3 are reported. For the sensors-loaded configuration, the results include the three cross-sections where the sensors insist and the two remaining markers (#1 and #2). Amplitudes vary from the values recorded at the first and at the last frame considered for the same recording; these end values can be safely assumed as being lower than the linear threshold or very close to it. 

**Figure 13:**
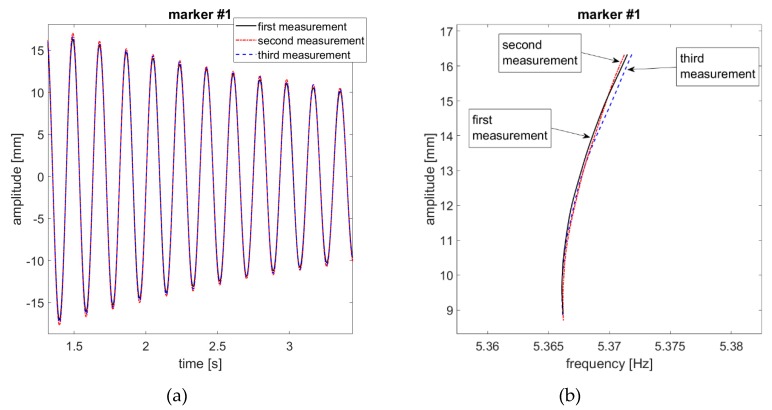
Example of three measured time histories (**a**) and the three backbone curves (**b**) extracted from them. Sensor-loaded conditions, marker #1, acquired at 500 fps.

Estimating the uncertainty of the measurements is not a trivial task, due to the high accuracy required in the estimation of the instantaneous amplitude and frequency. Yet this is imperative when proposing a novel measurement technique as done here, especially due to the high sensitivity of the geometric higher-order effects to even the slighter changes. Thus, to assess it properly, the BB curves have been computed with respect to three distinct video recordings with the same settings of the video camera and environmental conditions. Table 6 reports the beginning and end values of amplitude for the BB of Figure 12a,b; the ranges of frequencies (minimum to maximum) captured during the three recordings at the selected amplitudes are indicated as well. An exemplificatory case is depicted in Figure 13 (time series were aligned in order to be superposed).

**Figure 14:**
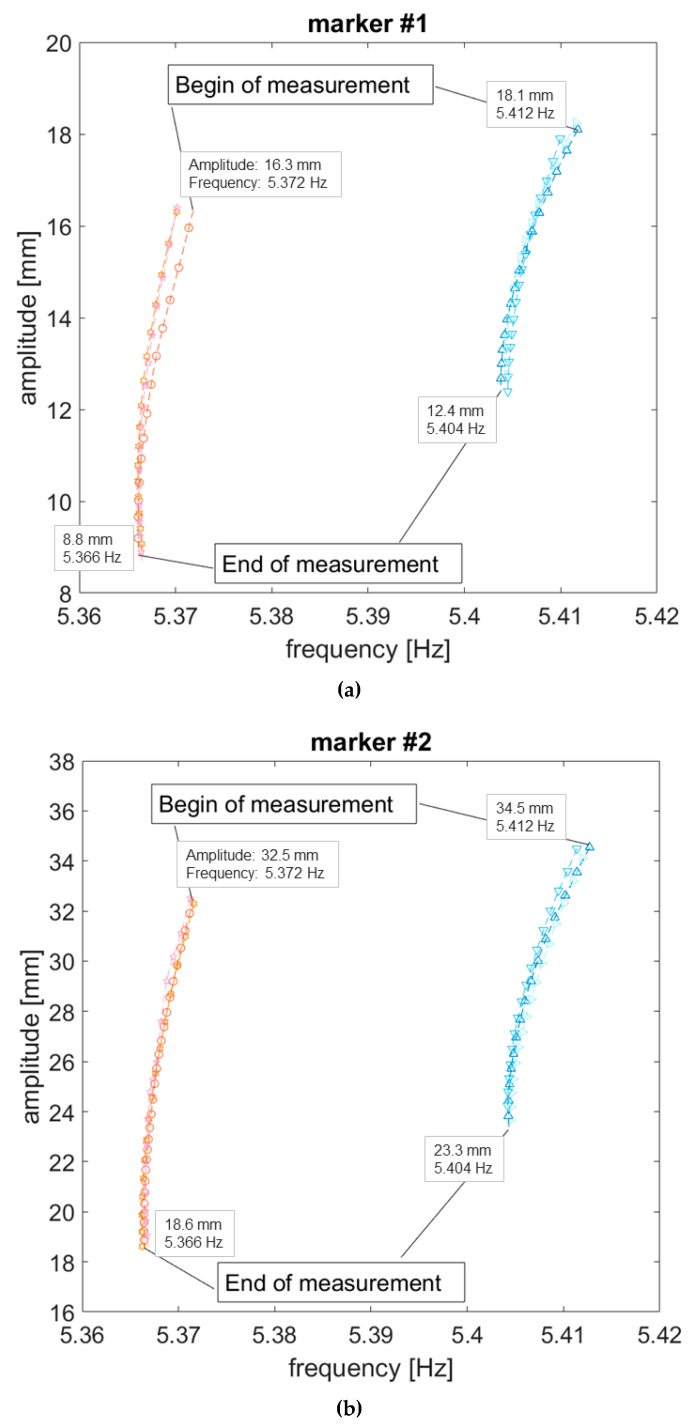
Comparison of backbone curves (loaded vs. unloaded condition) for (**a**) marker #1 and (**b**) marker #2, at their actual frequencies. Triangles in shades of blue correspond to the three measurements without sensors. The other red/pink markers refer to the three measurements with sensors attached.

The comparison between the sensors-loaded and the unloaded configuration can be summarised as follow. By focusing on the two shared markers #1 and #2, it can be noticed that the shift of the linear natural frequency between the unloaded and loaded linear resonance is quite limited (circa 0.4 Hz) yet evident and consistent with the theoretical expectations from an increase in the mass of the overall spar-sensors system (Figure 14) and with a linear FE model of the spar used as a benchmark. 

**Figure 15:**
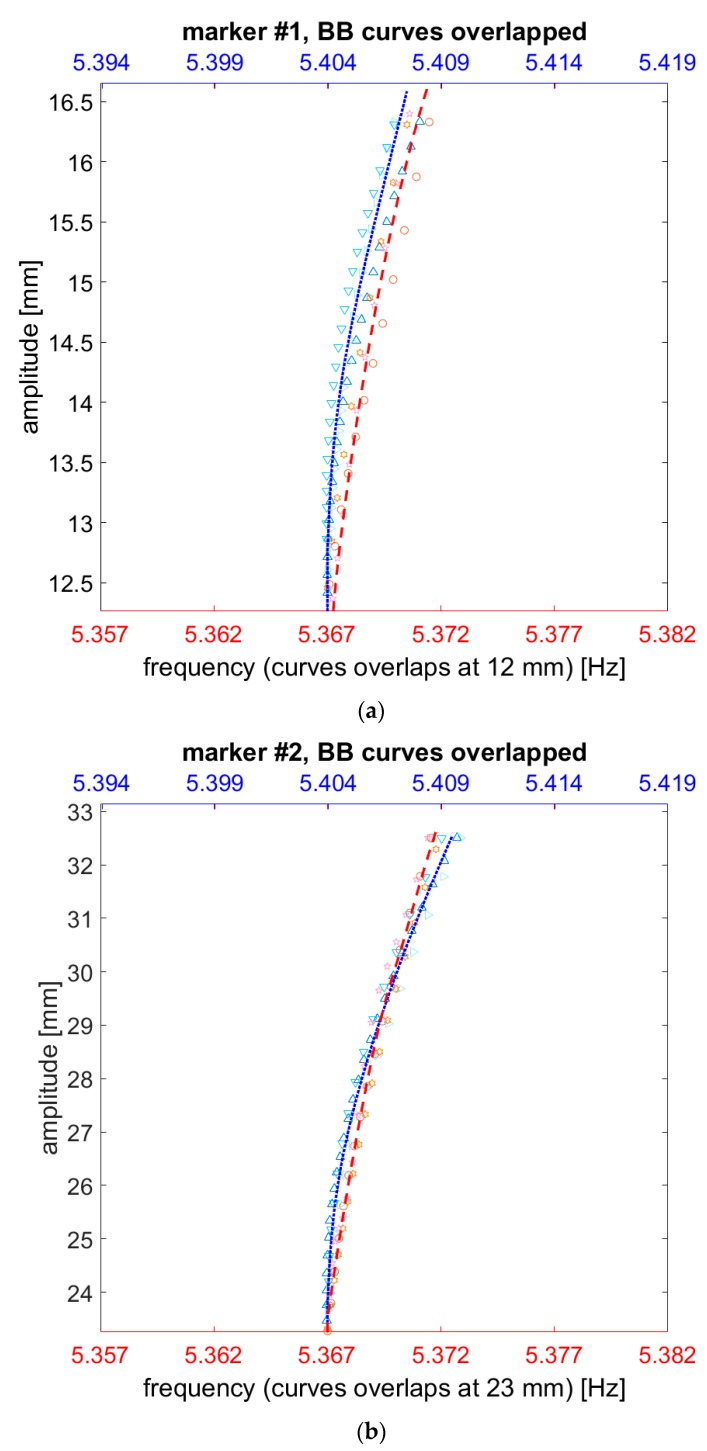
Comparison of backbone curves’ models (loaded vs. unloaded condition) for marker #1 (**a**) and marker #2 (**b**), overlapped. Triangles in shades of blue correspond to the three measurements without sensors. The other red/pink markers refer to the three measurements with sensors attached. The blue dotted and the red dashed lines indicate the models fitted on the respective data. The blue *x*-axis (**top**) refers to the unloaded condition; the red one (**bottom**) to the sensor-loaded spar.

By comparing the various measurements on the markers #1 and #2, it can be seen that at the same level of amplitude, due to the difference in the nonlinear hardening behaviour, the resonance frequencies become increasingly divergent. This variation of the nonlinear trend due to change in loading mass can be better visualised by overlapping the backbone curves at the lowest captured amplitude (here, at 15 mm and 25 mm, respectively, for marker #1 and #2) (Figure 15).

To define a single curve from multiple observations, an analytical formulation was developed, by means of a rational model fitted on the experimental data. A numerator of degree 5 and a denominator of degree 4 were found to adapt well to the experimental data for the range of frequencies and amplitudes of interest. The two pairs of models fitted over the data from marker #1 and #2 are indicated by the red and blue dashed lines in Figure 15; the estimators of the goodness of fit are reported in Table 7. 

The effects of sensor loading on the wing spar nonlinear dynamics during large-amplitude vibrations can be summed up as follows. First, for the same instantaneous frequency and for the same energy content of the input force, the unloaded HAR wing undergoes larger deflections, as easily foreseeable. Thus, it is more subject to both inertial and geometrical nonlinearities, making their competing effects more marked. Specifically, it seems that inertia-induced softening ‘slows down’ the tensile-stress induced hardening at relatively ‘low’ large deflections, with respect to the sensors-loaded condition (Figure 15). This may really also be caused by some additional linear or nonlinear damping effects (induced by incremented air drag or, more presumably, by the absence/presence of cables, tape and other elements attached to the airwing’s extrados). At larger deflections, however, the hardening effects of the unloaded configuration surpass their loaded counterparts. Unfortunately, the exact instant of the overtake was not captured for marker #1, but it can be guessed from the trends of the collected data and by comparing it with the behaviour of marker #2. 

The other main point observed here is that additional masses generated a (much) more sensible effect on the *nonlinear* response of the spar rather than on its linear natural frequencies, which were only slightly reduced.

These results found here are in accordance with what expected from theory and what has been recently described by [76], based on numerical results regarding reduced element stiffness. Indeed, a comparison is quite straightforward, as it is known that the effects of a reduction in stiffness are similar to the ones of an increase in mass, frequency-wise; that is, in fact, a strong limitation on the use of *linear* natural frequencies as damage detection features, as they easily confound mass-related effects with stiffness-induced ones. Thus, comparison between damaged–undamaged conditions can give some qualitative hints about loaded–unloaded configurations, and vice versa, at least in the linear domain. The similarities and/or differences between adding masses and reducing stiffness in the extension of the structural dynamics to the nonlinear case are still a matter of ongoing research instead.

## 7. Conclusions

Large-amplitude flexural vibrations and geometric nonlinearities are becoming issues of increasing interest as aeronautical manufacturers move to thinner and more bendable structural components. Future airplanes will require air wings that are able to undergo deflections orders of magnitudes greater than the current standards. However, sensor loading plays a crucial role in inspecting these nonlinear dynamics in laboratory and can be misleading.

Sensors’ masses alter the dynamic response of the inspected structure. This is often negligible, especially for massive, heavy structures, with small displacements and operating in linear conditions. However, these effects are generally nefarious for highly-flexible components, even if this fact is commonly overlooked; common practice is to consider the dynamic response of the structure–sensors whole system not too different from the unloaded structure alone. Raspberry ^®^ PI IMUs offer a minimally-invasive alternative, with negligible variation of the resting configuration $B_{0}$ in static conditions. Nevertheless, changes in linear and (especially) nonlinear dynamic response are still appreciable, while also other practical issues exist: for instance, large deflections can cause the sensors to detach. Noncontact approaches would therefore be much appreciated.

Other contactless alternatives to video acquisitions exist, but their use is hampered by intrinsic limitations. For example, multipoint laser tracking requires some sort of prior knowledge of the expected motion to be followed, and this is not feasible when these motions are unknown and/or difficulty predictable; moreover, any laser-based approach can only record displacements along the direction of the laser beam. By contrast, vertical and horizontal components of the curvilinear transverse motion can be easily isolated and investigated separately in two-dimensional frames.

Of particular importance is that analyses from video recordings can be performed theoretically at any point of the structure, producing a span-wise, full-field identification. The trajectories can be experimentally defined and easily approximated to define analytically a locus of points, which in this case turned out to be best fitted by parabolic equations. This can be used to experimentally investigate the exact dynamics of the structure’s large deflections.

In this work, the major focus was on a HAR prototype’s spar first mode, as it was the only one to experience displacements large enough to prompt a non-negligible hardening and to show evident nonlinearities for the given amplitude investigated here, which was the largest possible before risking irrecoverable damage to the specimen. 

For any selected point of interest, the amplitude–frequency curves which define the nonlinear continuation of the first mode were found; their corresponding motion was also experimentally defined and shown to be very well approximated by parabolic trajectories. Experimental evidence corroborates what was expected from the theory and often documented in the literature for a cantilever beam-like structure undergoing vibrations large enough to exceed its linear limit. A comparison was drawn between the sensors-loaded and unloaded structure. As expected, the unloaded structure underwent larger deflections at the same level of amplitude with respect to its unloaded counterpart; this made the nonlinear response of the first normal mode proportionally stiffer. It was also noticed that inertial loss causes a slight softening effect, which balances partially the geometric hardening. Some correlations between the nonlinear effects of added masses and the nonlinear effects of damage-induced stiffness reduction were also highlighted.

Some hints regarding the uncertainty of the BB curves estimation have been also reported, by repeating the measurements three times. This latter point is particularly interesting; nevertheless, it seems to have been rarely and not yet fully addressed in Literature. Authors are committed to going further deeper into the topic sooner.

This experimental study leaves plenty of room for improvement on the technical side. Extracted time histories proved to be accurate and dependable if a proper set-up is used, but the routine is not highly robust in non-optimal conditions; e.g., pixel brightness is known to be very sensitive to illumination. Some technical expedients, such as applying a coloured illumination (for instance, blue LED ultra-bright lights) and filtering out other colours, can increase results’ quality. The automatisation of the procedure will be necessary, as at the current state, extrapolating useful time histories from videos is time-consuming.

Further investigation is also needed specifically for the HAR wing here studied. Hopefully, further experimental evidence will be gathered and pieced together in a coherent theory for the effects of mass and stiffness local changes on the nonlinear dynamic response of the air wing spar.

## Figures and Tables

**Table 1:** Geometrical and mechanical properties of the wing spar.

Density ρ	2850	kg/m^3^
Young’s Modulus *E*	$7.31\times 10^{10}$	Pa
Poisson Ratio ν	0.33	-
Free length (clamp to tip) $l_{tip}$	706	mm
Thickness *t*	2	mm
Max width at clamped section $b_{\max}$	180.00	mm
Mid-length width at the section of changing tampering (l=258 mm) $b_{l=258}$	56.10	mm
Min width at tip section $b_{\min}$	17.04	mm

**Table 2:** Technical details of the instrumentation and parameters set.

**Raspberry PI ^®^*IMUs***	
**Sampling Frequency**	100 Hz
Sensor Type	MPU-6050 InvenSense *™* sensors (3 DoF accelerometer + 3DoF gyroscope)
Accelerometer Full Scale Range & Sensitivity	±16 g; 2048 LSB/g
**Olympus ^®^*I-speed 3 ™***	
Frame Rate	500 fps/1000 fps (max available: 150,000 fps)
Focus	Automatic
Sensor Type	CMOS sensor
Pixel Resolution	1280 (W) × 1024 (H)
Shutter Time & Type	1 μs, global exposure, electronic
Internal Memory	8 GB (max 4897 frames in memory at 1280 × 1024 resolution)
**Polytec ^®^*OFV-505 Sensor Head ™***	
Sampling Frequency	2000 Hz
Focus	Automatic
Laser Type & Wavelength	Helium Neon, 633 nm

**Table 3:** First linear natural frequency for the unloaded and sensor-loaded conditions.

	First natural frequency. Frequency resolution: Δf=0.0034 Hz (% of the unloaded condition)
No sensor attached (unloaded except the 4 markers)	5.403 Hz (~100%)
Raspberry PI ^®^ 4 IMUs + cables, tape and 2 markers	5.366 Hz (99.27%)

**Table 4:** Settling times for the unloaded air wing spar.

	T_s10%_
	[s]
1st natural frequency	12.43
2nd natural frequency	4.23
3rd natural frequency	1.44

**Table 5:** Goodness-of-fit statistics for parabolas fitted over experimental data.

	Marker #1	Marker #2	Marker #3
$p_{1}$	−0.001601	−0.0009294	−0.0005539
95% confidence bounds	−0.001648, −0.001555	−0.0009355, −0.0009234	−0.0005552, −0.0005527
*SSE*	149.2882	22.6939	28.4734
$R^{2}$	0.7085	0.9499	0.9975
${\bar{R}}^{2}$	0.7084	0.9499	0.9975
*RMSE*	0.1747	0.0681	0.0763
*DFE*	4892	4892	4892

**Table 6:** Beginning and end amplitude for the targets in unloaded and loaded conditions.

Position *Y* [mm]	*Unloaded Beam*			*Sensors-Loaded Beam*		
	Target	End Amplitude	Begin Amplitude	Target	End Amplitude	Begin Amplitude
		(Corresponding Instantaneous Frequency Range in Brackets)			(Corresponding Instantaneous Frequency Range in Brackets)	
		[mm] (Hz)	[mm] (Hz)		[mm] (Hz)	[mm] (Hz)
180	Marker #0	7.5 (5.4041–5.4048)	11.0 (5.4097–5.4119)	-	-	-
200	-	-	-	IMUs #1 & #2	5.2 (5.3660–5.3665)	9.3 (5.3715–5.3719)
250	-	-	-	IMU #4	8.0 (5.3660–5.3665)	15.1 (5.3706–5.3716)
260	Marker #1	12.4 (5.4038–5.4045)	18.1 (5.4099–5.4118)	Marker #1	8.8 (5.3661–5.3662)	16.3 (5.3711–5.3716)
390	Marker #2	23.3 (5.4041–5.4043)	34.5 (5.4114–5.4117)	Marker #2	18.6 (5.3662–5.3668)	32.5 (5.3712–5.3716)
450	-	-	-	IMU #3	24.3 (5.3662–5.3663)	43.2 (5.3715–5.3717)
600	Marker #3	45.9 (5.4044–5.4045)	67.9 (5.4112–5.4116)	-	-	-

**Table 7:** Models fitted over three measurements: Goodness of the fitted models.

	*No Sensors*		*With Sensors*	
	Marker #1	Marker #2	Marker #1	Marker #2
*SSE*	0.9222	5.598	2.606	8.041
$R^{2}$	0.9996	0.9997	0.9994	0.9995
${\bar{R}}^{2}$	0.9996	0.9997	0.9994	0.9995
*RMSE*	0.01895	0.04035	0.03438	0.05973

## Abbreviations

The following abbreviations are used in this manuscript:

LMA	linear modal analysis
NLMA	nonlinear modal analysis
HAR	high aspect ratio
EB	Euler–Bernoulli
NNM	nonlinear normal mode
RS	reference system
BB	backbone curve
RDM	resonance decay method
LDV	laser Doppler vibrometer
IMU	inertial measurement unit
ZC	zero crossing
PE	peak envelope
HF	Hilbert transform
ODS	operational deflection shapes
TH	time history
